# Trauma quality indicators: internationally approved core factors for trauma management quality evaluation

**DOI:** 10.1186/s13017-021-00350-7

**Published:** 2021-02-23

**Authors:** Federico Coccolini, Yoram Kluger, Ernest E. Moore, Ronald V. Maier, Raul Coimbra, Carlos Ordoñez, Rao Ivatury, Andrew W. Kirkpatrick, Walter Biffl, Massimo Sartelli, Andreas Hecker, Luca Ansaloni, Ari Leppaniemi, Viktor Reva, Ian Civil, Felipe Vega, Massimo Chiarugi, Alain Chichom-Mefire, Boris Sakakushev, Andrew Peitzman, Osvaldo Chiara, Fikri Abu-Zidan, Marc Maegele, Mario Miccoli, Mircea Chirica, Vladimir Khokha, Michael Sugrue, Gustavo P. Fraga, Yasuhiro Otomo, Gian Luca Baiocchi, Fausto Catena, the WSES Trauma Quality Indicators Expert Panel, Zygimantas Kuliesius, Luigi Conti, Agron Dogjani, Jae Gil Lee, Heitor Consani, Domenico Russello, Marina Bortul, Teresa Gimenez Maurel, Hossein Samadi Kafil, Harissou Adamou, Vasilescu Alin, Umberto Robustelli, Norio Sato, Charalampos Seretis, Martha Quiodettis, Carlos Augusto Gomes, Victor Kong, Andee Dzulkarnaen Zakaria, Ali Guner, Mahir Gachabayov, Sharfuddin Chowdhury, Francesco Pata, Alberto Garcia, Miran Rems, Koray Das, J. G. Riedel, Konstantinos Lasithiotakis, Ruslan Sydorchuk, Larysa Sydorchuk, Eftychios Lostoridis, Alexander Buia, Michael McFarlane, Renzo Ciani, Virginia María Durán Muñoz-Cruzado, Dario Tartaglia, Orestis Ioannidis, Måns Muhrbeck, Martin Reicher, Francesco Roscio, Marco Ceresoli, Dimitrios Tsiftsis, Alfie Kavalakat, Tadeja Pintar, George Georgiou, Gabriele Ricci, Rajashekar Mohan, Sten Saar, Isidoro Di Carlo, Arda Isik, Ali Yasen Yasen Mohamed Ahmed, Ricardo Alessandro Teixeira Gonsaga, Fabrizio Sammartano, Luis Tallon-Aguilar, Tomohisa Shoko, Jeremy Hsu, Yoshiro Kobe, Christian Galatioto Luigi Romeo, Mauro Podda, Andrea Mingoli, Rafael Castro Delgado, Gerald Ekwen, Vanlander Aude, Carles Olona, Paolo Boati, Stefano Magnone, Massimo Capaldi, Miklosh Bala, Edoardo Picetti, Ionut Negoi, Kenneth Y. Y. Kok, Asri Che Jusoh, Bruno Amato, Gabriela Elisa Nita, Andrew de Beaux, Zaza Demetrashvili, R. Justin Davies, Jae Il Kim, André Pereira, Luca Fattori, Ciro Paolillo, Wagih Ghannam, Fernando Machado Rodriguez, Luca Berardi, Maria Gioffrè Florio, Matthias Hecker, Vincent Dubuisson, Donal B. O’Connor, Nicola De’Angelis, Ivan Dobrić, Damien Massalou, Per Örtenwall, Emmanouil Pikoulis, Bakarne Ugarte-Sierra, W. P. Zuidema, Aristotelis Kechagias, Sanjay Marwah, Andrey Litvin, Ioannis Nikolopoulos, Antonio Pesce, Selman Uranues, Davide Luppi, Sascha Flohe, Aleix Martínez-Pérez, Manuel Lorenzo, Luigi Branca Vergano, Mario Manca, Paolo Malacarne, Hayato Kurihara, Sandy Widder, Marsia Pucciarelli, Fabio Monzani, Pietro Brambillasca, Davide Corbella, Ferdinando Agresta, Lynne Moore, Luis Antonio Buonomo, Amos O. Adeleye, Dennis Kim, Massimiliano Veroux, Timothy Craig Hardcastle, Salomone Di Saverio, Alfonso Recordare, Ines Rubio-Perez, Sergey Shlyapnikov, Razrim Rahim, Gustavo Miguel Machain Vega, Kessel Boris, Robert Sawyer, Oussama Baraket, Kjetil Soreide, Clemens Weber, Chen-June Seak, Simon Herman, Emiliano Gamberini, Silvia Costa, Gualtiero Mazzocconi, Edgard Lozada, Dimitrios Manatakis, Varut Lohsiriwat, Adamu Ahmed, Bahaa Elbery, Guido Alberto Massimo Tiberio, Massimo Santini, Luca Mellace, Cathrine Harstad Enoksen, Piotr Major, Dario Parini, Mario Improta, Paola Fugazzola, Silvia Pini, Gaetano Liberti, Costanza Martino, Lorenzo Cobianchi, Gabriele Canzi, Enrico Cicuttin, Jakub Kenig, Mauro Zago, Sandro Giannessi, Michelangelo Scaglione, Eugenio Orsitto, Roberto Cioni, Lorenzo Ghiadoni, Francesco Menichetti, Vanni Agnoletti, Gabriele Sganga, Paolo Prosperi, Franco Roviello, Paolo De Paolis, Giovanni Gordini, Francesco Forfori, Paolo Ruscelli, Francesco Gabrielli, Adolfo Puglisi, Andrea Bertolucci, Santino Marchi, Massimo Bellini, Sergio Casagli, Belinda De Simone, Fabio Carmassi, Stefano Marchetti, Marco Accorsini, Camilla Cremonini, Federica Morelli

**Affiliations:** 1 General Emergency and Trauma Surgery Department, Pisa University Hospital, Via Paradisa, 2, 56124 Pisa, Italy; 2 Division of General Surgery, Rambam Health Care Campus, Haifa, Israel; 3 Ernest E Moore Shock Trauma Center, Denver Health, Denver, CO USA; 4 Department of Surgery, Harborview Medical Center, University of Washington, Seattle, WA USA; 5 Riverside University Health System, Riverside, CA USA; 6 Division of Trauma and Acute Care Surgery, Fundación Valle del Lili, Cali, Colombia; 7 VCU Medical Center, Richmond, VA USA; 8 General, Acute Care, Abdominal Wall Reconstruction, and Trauma Surgery, Foothills Medical Centre, Calgary, Canada; 9 Department of Trauma and Acute Care Surgery, Scripps Memorial Hospital La Jolla, La Jolla, San Diego, CA USA; 10 General and Emergency Surgery, Macerata Hospital, Macerata, Italy; 11 Department of General and Thoracic Surgery, University Hospital of Giessen, Giessen, Germany; 12 General, Emergency and Trauma Surgery Department, Bufalini Hospital, Cesena, Italy; 13 Abdominal Center, Helsinki University Hospital, Helsinki, Finland; 14 Department of War Surgery, Kirov Military Medical Academy, Saint-Petersburg, Russia; 15 General and Emergency Surgery Dept., Auckland City Hospital, Auckland, New Zealand; 16 Department of Surgery, Hospital Angeles Lomas, Mexico City, Mexico; 17 Faculty of Health Sciences, University of Buea, Buea, Cameroon; 18 Douala Gynaeco-Obstetric and Pediatric Hospital, Douala, Cameroon; 19 General Surgery Department, University Hospital St George, Plovdiv, Bulgaria; 20 Department of Surgery, University of Pittsburgh School of Medicine, Pittsburgh, USA; 21 Trauma Team and General Surgery, ASST Niguarda, Milan, Italy; 22 Department of Surgery, College of Medicine and Health Sciences, UAE University, Al-Ain, United Arab Emirates; 23 Department of Trauma and Orthopedic Surgery, Cologne-Merheim Medical Center (CMMC), University Witten/Herdecke (UW/H), Cologne, Germany; 24 Statistic Dept., Pisa University, Pisa, Italy; 25 Centre Hospitalier Universitaire Grenoble Alpes, Grenoble, France; 26 Department of Emergency Surgery, City Hospital, Mozyr, Belarus; 27 General Surgery Dept., Letterkenny Hospital, Letterkenny, Ireland; 28 Division of Trauma Surgery, School of Medical Sciences, University of Campinas, Campinas, Brazil; 29 Trauma and Acute Critical Care Center, Tokyo Medical and Dental University Hospital, Tokyo, Japan; 30 General Surgery, Brescia University Hospital, Brescia, Italy; 31 Emergency and Trauma Surgery, Parma University Hospital, Parma, Italy

**Keywords:** Performance, Product, Morbidity, Mortality, System, Analysis, Outcome, Data, Planning, World

## Abstract

**Introduction:**

Quality in medical care must be measured in order to be improved. Trauma management is part of health care, and by definition, it must be checked constantly. The only way to measure quality and outcomes is to systematically accrue data and analyze them.

**Material and methods:**

A systematic revision of the literature about quality indicators in trauma associated to an international consensus conference

**Results:**

An internationally approved base core set of 82 trauma quality indicators was obtained: Indicators were divided into 6 fields: prevention, structure, process, outcome, post-traumatic management, and society integrational effects.

**Conclusion:**

Present trauma quality indicator core set represents the result of an international effort aiming to provide a useful tool in quality evaluation and improvement. Further improvement may only be possible through international trauma registry development. This will allow for huge international data accrual permitting to evaluate results and compare outcomes.

## Background

Quality in medical care must be measured in order to be improved. Trauma management is part of health care, and by definition, it must be checked constantly. The only way to measure quality and outcomes is to systematically accrue data and analyze them. However, one of the main issues encountered in this activity is the difficulty to obtain complete and affordable dataset. Health care systems as well as trauma systems are different. They are differently organized around the world; discrepancies exist between them. The profound differences in organizational models may reflect even in outcomes. The necessity to evaluate the quality of care in a local, national, and even international scale has been progressively considered more necessary in the last decades. Quality of care is characterized as “the degree to which health services for individuals and populations increase the likelihood of desired health outcomes and are consistent with the current professional knowledge” [1]. Measurement and feedback of performance are integral to the concept of a system of care [2, 3]. Since the early 1970s, the evidence of several deaths due to suboptimal trauma care in the USA has led to the development of structured trauma systems [4]. With the development of organizational models, the number of preventable deaths has progressively decreased [2]. Quality improvement evaluates the performance of both individual providers and the systems in which they work [1].

Evaluation of quality of the service offered by health systems may be measured with quality indicators (QI).

QI are performance measures designed to compare actual care against ideal criteria for the purposes of quality measurement, benchmarking, and identifying potential opportunities for improvement [5].

The US national system was the first in developing a structured trauma quality indicators (TQI) list and in providing several tools in order to continuously check and improve results. At present, many different TQI sets exist. However, concomitant existing significant variations in the utilization of indicators and limited evidence to support the use of specific indicators over others do not allow for an exchange in TQI within the different systems [5]. In fact, around the world, trauma systems are at different points in the organizational progression. TQI list generally adopted in a system cannot be entirely applied in a different one. Actually, no clearly defined and internationally approved TQI sets exist. However, a core set of universally applicable TQI that may be transversally adopted by all trauma systems is needed. Subcategories of indicators may then be elaborated and tailored according to dedicated system analysis.

The aim of this paper is to present a list of internationally approved core items for trauma management quality evaluation.

## Material and methods

A systematic revision of the literature about QI for evaluating trauma care was conducted. Researches were done on MEDLINE, Embase, CINAHL, Cochrane Database of Systematic Reviews, Cochrane Database of Abstracts of Reviews of Effects, and Cochrane Central Register of Controlled Trials from the earliest available date through May 31, 2019. To increase the sensitivity of the search, the grey literature and select journals by hand were investigated, reference lists to identify additional studies were reviewed, and experts in the field were contacted. Moreover, websites of the major surgical and critical care societies worldwide were investigated for obtaining QI (American College of Surgeons, American Association for the Surgery of Trauma, Eastern Association for the Surgery of Trauma, Western Trauma Association, American Trauma Society, International Trauma Anesthesia, and Critical Care Society, British Trauma Society, Panamerican Trauma Society, Trauma Association of Canada, European Society for Trauma and Emergency Surgery, Australasian Trauma Society, Orthopedic Trauma Association, Trauma.org, the Society of Trauma Nurses). To further enlarge the research, also the main web search engines were utilized (i.e., Google, Yahoo, Bing, and Baidu) using the following search terms: trauma, quality, indicator, and injury.

All articles identifying and/or proposing 1 or more QI focusing on prehospital care, hospital care, posthospital care, or secondary injury prevention were considered.

Moreover, main world trauma centers’ TQI lists were analyzed. All the identified QI lists were then analyzed in order to summarize all retrieved indicators.

Once all the QI were summarized, an international expert panel web-based consensus survey was done to obtain a balanced QI list. Two hundred experts from all the 5 continents and from all the 6 WHO regions were asked to express their evaluation of importance (0–10 marks, where 0 was not relevant and 10 was very important) about all the proposed QI. Items with ≥ 70% of preferences to values 8 to 10 have been accepted as important and passed through the next steps. During the survey, expert panel components had the opportunity to suggest further quality indicators they consider important and not present in the proposed list.

Results of the survey were analyzed and discussed during an international event in Pisa, Italy, on September 27, 2019. Then, results of discussion were diffused for a further international round of evaluation and discussion between international panels of recognized experts in the field. Through subsequent rounds of evaluation, in a modified Delphi process, the manuscript reached the definitive version together with the definitive TQI core list.

## Results

After systematic reviews of the existing literature about TQI and the trauma center/society protocol and TQI lists, a total of 1288 indicators were obtained. After analysis and elimination of duplicate QI or integration of the similar ones into a single comprehensive indicator, 89 were proposed for international evaluation. After international round, 82 were considered to be included into the definitive list (Tables 1, 2, 3, and 4).

**Table 1:** Prevention and structure indicators

Category	Subcategory	Indicators	Patients
Prevention		Activity to prevent and diffuse trauma risks and effect perception	All patients
		Measurement of injury risk perception and behavioral changes following sensibilization programs	All patients
		Psychological consequences in observers	All patients
		Copycat event prevention	All patients
		Direct medical cost quantification	All patients
		Indirect cost quantification	All patients
Structure	Center preparedness	Presence of data registry	All patients
		Staff training requirements	All patients

**Table 2:** Process indicators (*TTA* Trauma Team Activation, *GCS* Glasgow Coma Scale, *TBI* traumatic brain injury, *ED* emergency department, *AIS* Abbreviated Injury Scale, *ISS* Injury Severity Score, *CT* computed tomography, *TEG* tromboelastography, *ROTEM* rotational thromboelastometry, *ICU* intensive care unit, *EX-LAP* explorative laparotomy, *SBP* systolic blood pressure, *OR* operating room, *E-FAST* extended focused assessment with sonography in trauma, *REBOA* resuscitative endovascular balloon occlusion of the aorta, *CNS* central nervous system)

Category	Subcategory	Indicator	Patients
Process	**Triage/prehospital**	Time to first medical contact (on scene)	All patients
		Prehospital time	ISS > 16
		Time to definitive trauma center	All patients
		Acute pain management	Patients with documented pain assessment
		Intubation of unconscious patients	Prehospital GCS < 9
		Pelvic binder in pelvic fracture	Mechanically and/or hemodynamically unstable pelvic fractures (AIS 3-5)
		Field triage rate (undertriage)	All patients
		Patient in shock with documented blood pressure who dies with no Emerg. Dept. thoracotomy or REBOA placement	Patients died in ER arrived with a documented blood pressure
	**Emergency dept. management**	Trauma Team Activation (TTA)	Patients requiring TTA for whom TTA was activated
		Airway secured in ED for patients with GCS <9	Patients with GCS < 9
		Tracheal intubation (GCS<9)	Patients with GCS < 9
		Adequate rewarming measures for hypothermia (temperature ≤ 35 °C)	Patients admitted to a trauma center
		Operative management of patients with an abdominal gunshot wound	Patients with a penetrating abdominal injury by firearm
		Tetanus prophylaxis	All patients with exposed soft tissues
		Antibiotics for open fractures	Number of patients with an open fracture receiving an antimicrobial agent within 1 h of hospital arrival
		Time to cranial CT for patients with GCS < 14	GCS < 14
		Patient with GCS < 13 has a head CT within 4 h of arrival in ED	Adult TBI: GCS < 13; pediatric TBI: GCS < 12
		Time to CT scan from ED admission	ED patients with blunt force injuries AND trauma team activation (TTA) OR ED documented GCS < 9, receiving CT scan within 1 h of ED arrival
		E-FAST in patient without CT	Patients without CT
		Blood analysis performed/BE documented	All patients
		Coagulation test (TEG/ROTEM)	All patients with active bleeding
		ED stay > 1 h for patients with GCS < 9 or intubated (level I/II)	TBI patients with GCS ≥ 4 or ≤ 10 in a level I/II trauma center
		ED stay > 1 h for patients admitted to ICU or OR	TBI patients with GCS ≥ 4 or ≤ 8 or intubated in a level I/II trauma center
		Massive trasfusion protocol activation	Patients with active bleeding and signs of shock
		Time to start of blood transfusion	Patients with at least one unit transfused
		Orthopedic response time > 30 min in emergent case	Patients with orthopedic trauma
		Unplanned ICU admission	Patients primarily admitted to ward then moved to ICU
	**Surgical management**	Definitive bleeding control (in patients with PTM)	All patients age 18 years and older with an injury diagnosis AND prescribed a massive transfusion who receive attempted definitive bleeding control (laparotomy, thoracotomy, percutaneous therapy) within 30 min of the massive transfusion prescription
	**Trauma**	Time to first emergency surgery	Operated patients
		Delay to OR-EX-LAP (> 2 h): trauma	Operated patients
		Time to laparotomy < 1 h for patients with a proven intra-abdominal bleeding causing hypotension	SBP < 90 or requires > 4 units of packed red blood cells in the first hour for hemorrhage due to injury
		Time to surgery in patients with shock	SBP < 90
		Patients with bleeding pelvic fracture who die within 60 min from ED arrival without preperitoneal pelvic packing or REBOA placement	Patients with bleeding pelvic fracture
	**Neurosurgical**	Time to surgical brain decompression	TBI with indication for decompression
		Patients with epidural or subdural hematoma receiving craniotomy > 4 h after arrival	Patients with epidural or subdural hematoma
		Enteral or parenteral feeding for severe head injury patients < 7 days post-injury	TBI patients with GCS ≤ 10
		Failure monitoring of intracranial pressure in severe TBI with pathological CT finding	Severe TBI
	**Orthopedic**	Open fracture grade 3 to OR > 8 h	Open fracture grade 3
		Open long bone fracture surgery < 6 h	Open fracture of the tibia, fibula, humerus, radius, or ulna
		Patient with pelvic fracture and hemodynamic instability on ED arrival with provisional stabilization of pelvic ring fracture within 12 h from arrival at the trauma center	Patients with SBP < 90 or requiring > 4 units of packed red blood cells in the first hour
		Open fracture grade 1 or 2 to OR >16 h	Open fracture grade 1 or 2
		Open fractures—stabilized > 24 h	Long bones open fractures
	**Vascular**	Ischemic limb revascularized < 6 h	Ischemic limb following vascular trauma
		Time to restore perfusion	Ischemic limb following vascular trauma
		Deep vein thrombosis prophylaxis (within 24 h) in immobile patients	Patients immobilized ≥ 24 h (without CNS bleeds or spine/CNS surgery within 24 h)
		Patients who experienced limb amputation without previous vascular shunt placement	Patients with limb amputation

**Table 3:** Outcome, post-traumatic management, and society integrational effect indicators (*VAE* ventilator-associated events, *TBI* traumatic brain injury, *ED* emergency department, *ICU* intensive care unit, *OR* operating room)

Category	Subcategory	Indicator	Patients
Outcome	Admission data	ICU lenght of stay	Patients admitted to ICU
		Lenght of stay	All patients
		Ventilator-associated events (VAE)	All patients
	Adverse events (according to Clavien-Dindo classification)	Complications during hospital stay	All patients
		Pulmonary embolus	All patients
	Mortality	Mortality rate	Admitted patients
		Death < 48 h after arrival	All patients
		Deaths >1 h after arrival occur on ward (not in ED)	Vital signs on arrival
		Death > 48 h after arrival	All patients
		Mortality in severe TBI	Severe TBI
		Penetrating injury mortality	Patients with penetrating injury
		Blunt multisystem injury mortality	Patients with multisystem injury
		Blunt single-system mortality	Patients with single-system injury
		TBI deaths > 3 h following arrival in level III/IV center	TBI with GCS >12 and max head AIS > max AIS in other anatomic regions
	Failure to rescue (severe)	Patients died with unsolved severe complication	Patients who died among those with Clavien-Dindo grades 3–5 complications
	Functional outcome	Evaluation of patient functional status (at hospital)	All patients
	Outcomes review	Peer review of trauma deaths to evaluate quality of care and determine whether the death was potentially preventable	Dead patients
	Early post-op events	Tertiary survey	All patients
		Unexpected return to OR	All operated patients with no ongoing damage control surgery
Post-traumatic management		Long-term physical disability facilities/support	All patients
		Psychological disability facilities/support	All patients
		Behavioral change and secondary health loss quantification	All patients
		Tangible costs quantification	All patients
		Intangible costs quantification	All patients
Society integrational effects		Observer consequences evaluation/support	All patients
		Carer consequences evaluation/support	All patients
		Dependent consequence evaluation/support	All patients

**Table 4:** Secondary analysis of primary indicators

1. Error in management	
2. Error in judgment, deviation for internal protocols	
3. Error in diagnosis	
4. Error in technique	
5. Provider errors:	
• Treatment below the standard of care	
• Missed injuries	
• Error in prioritizing order of work up	
• Missing trauma scores: RTS, ISS, NISS, TRISS, etc.	
6. Morbidity and mortality rates in frail patients (i.e., elderly or transplanted)	

Average agreement was of 97% within the different experts about the different QI.

**Fig. 1:**
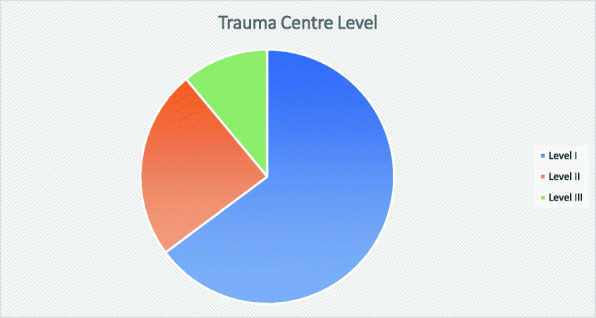
Trauma center level distribution of international experts

**Fig. 2:**
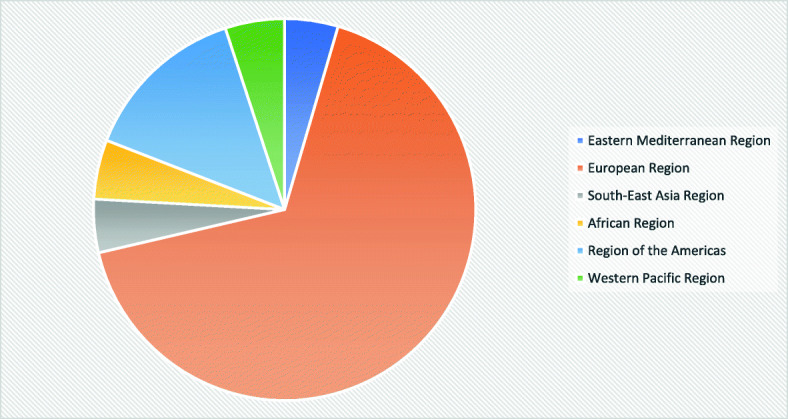
Expert distribution according to the World Health Organization (WHO) regions

**Fig. 3:**
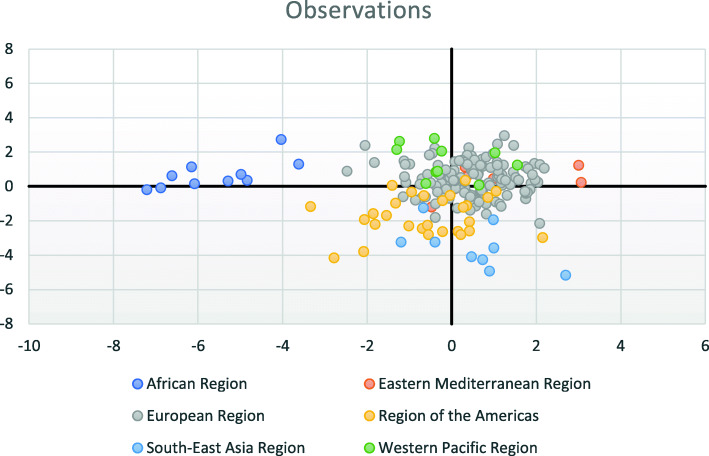
Differences in perceiving the importance of the different items for the different answers according to the WHO region

**Fig. 4:**
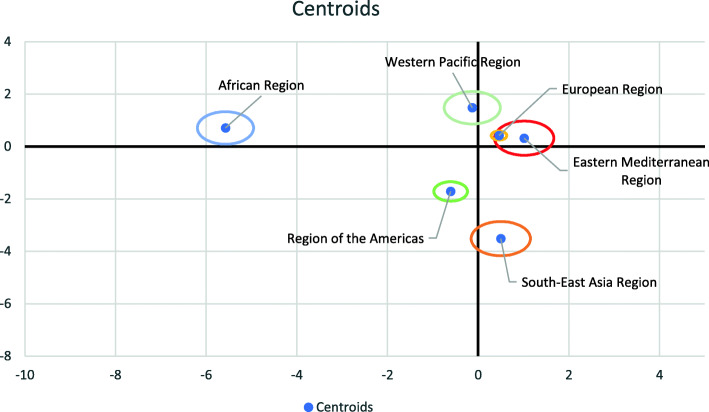
Centroid distribution of the differences in perceiving the importance of the different items for the different answers according to the WHO region

Participating centers and surgeon distribution across the different hospitals in the World Health Organization (WHO) regions are presented in Figs. 1 and 2. Answers were analyzed, and the distributions of the importance given to the different indicators have been reported in Figs. 3 and 4 showing some variation within the different the WHO regions.

The different regions showed homogeneous differences in perceiving the importance of the different items for the different answers (Fig. 3) and for the centroid of the average of the various answers (Fig. 4).

Categories into which TQI have been divided are as follows:

- Prevention
- Structure
- Process
- Outcome
- Post-traumatic management
- Society integrational effects

## Discussion

According to the WHO definitions, quality comprises three elements: structure, process, and outcome [1].

Structure refers to stable, material characteristics (infrastructure, tools, technology) and the resources of the organizations that provide care and the financing of care (levels of funding, staffing, training, skills, payment schemes, incentives) [1].

Process is the interaction between caregivers and patients during which structural inputs from the health care system are transformed into health outcomes. The process is the actual provision of medical care to the patient [1].

Outcomes can be measured in terms of health status, deaths, or disability-adjusted life years—a measure that encompasses the morbidity and mortality of patients or groups of patients. Outcomes also include patient satisfaction or patient response to the health care system [1].

At present, however, trauma system evolution must take into consideration the necessity to relate the system to the context into which it operates. For this reason, quality evaluation must comprise some more aspects influenced by and influencing the trauma patient’s management.

Present manuscript aims to answer to the recognized necessity of an international agreement about a QI core set. Quality improvement is mainly a behavioral change, and it is impossible to change if no shared and agreed points exist. Shared and largely approved and agreed-on QI are needed to improve quality not in a competitive view but in a reciprocal improvement behavior. It is not possible to proceed with a transparent, explicit, systematic, data-driven performance measurement if there is no agreement upon indicators and measures. A very high number of existing proposed TQI have been searched, reported, and resumed. For sure, search may not have been exhaustive, despite the evaluation of multiple databases using comprehensive research strategies and imposing no language restrictions. As a counterpart, the very high number of redundant indicators clearly shows how it approximated the completeness. We can assume that very few eventual other QI may have been not considered.

Present paper demonstrated that a common set of clearly defined, evidence-based, broadly accepted trauma QIs does not exist. A large group of heterogeneous indicators are diffusely and non-homogeneously utilized. Moreover, the vision and perception of TQI across the world is widely different as clearly shown in Figs. 3 and 4. This different perception is the reflection of different cultural and organizational models, and at the same time, it results in slightly different priorities. However, present international effort aims also to balance the differences in a shared TQI in order to promote intersystem comparison and improvement.

One of the main factors emerging from the analysis is the imbalance existing between QIs evaluating prehospital, in-hospital, and posthospital management. In fact, current literature universally focused on in-hospital phase of trauma care. Very few QIs are dedicated to the analysis of the pre- and posthospital phases. This reflects the lack in organizational systems, indifferently from the WHO region and from the resources of the system. This may be due to a disconnect between the professional figures reflecting on the three phases of trauma management. The in-hospital phase is diffusely considered the most important. For this reason, organizational efforts are maximized in this part with very few resources dedicated to the others. However, it should be stressed as the pre- and posthospital phases may strongly impair the effectiveness of the in-hospital trauma management. Lastly, prevention phase is not considered nor evaluated at all.

Donabedian stratification of healthcare QIs into structure, process, and outcome evaluation is valid and diffusely accepted [6–8]. However, as trauma involves more than the hospital and may impact on multiple different levels of the sanitary and economical systems, its quality management evaluation should encompass more than the already defined three key-points. It must consider the system in which the “structure” is included in, and international trauma registries must consider obtaining data even regarding socio-economical setting together with the performance of the specific hospital/system.

This paper proposes a six level stratification of TQI: prevention, structure, process, outcome, post-traumatic management, and society integrational effects.

Quality measures other than mere hospital morbidity and mortality and management process are strongly needed to evaluate the real outcome dimensions referring to trauma prevention, health-related quality-of-life, psychosocial impact of the injury, etc. with the aim of providing a more refined specificity for all the different components of patient care.

All these phases reflect even direct and indirect costs that may be even very important in a national and international view. For these reasons, they should also be included into trauma system quality evaluation. Cost evaluation however should be done at a local or national level. International cost comparison may be impossible or at least useless due to vastly different organizational/legal/economical models.

Lastly, a need to improve the science behind the development, validation, and use of indicators is urgent.

## Conclusion

Present trauma quality indicator core set represents the result of an international effort aiming to provide a useful tool in quality evaluation and improvement. Further improvement may only be possible through international trauma registry development. This will allow for huge international data accrual permitting to evaluate results and compare outcomes.

## Data Availability

Not applicable

## Abbreviations

QI: Quality indicators
TQI: Trauma quality indicators
WHO: World Health Organization
TTA: Trauma Team Activation
GCS: Glasgow Coma Scale
TBI: Traumatic brain injury
ED: Emergency department
AIS: Abbreviated Injury Scale
ISS: Injury Severity Score
CT: Computed tomography
TEG: Tromboelastography
ROTEM: Rotational thromboelastometry
ICU: Intensive care unit
EX-LAP: Explorative laparotomy
SBP: Systolic blood pressure
OR: Operating room
E-FAST: Extended focused assessment with sonography in trauma
REBOA: Resuscitative endovascular balloon occlusion of the aorta
CNS: Central nervous system
VAE: Ventilator-associated events
