# Correction: Trauma quality indicators: internationally approved core factors for trauma management quality evaluation

**DOI:** 10.1186/s13017-025-00577-8

**Published:** 2025-04-07

**Authors:** Federico Coccolini, Yoram Kluger, Ernest E. Moore, Ronald V. Maier, Raul Coimbra, Carlos Ordoñez, Rao Ivatury, Andrew W. Kirkpatrick, Walter Biffl, Massimo Sartelli, Andreas Hecker, Luca Ansaloni, Ari Leppaniemi, Viktor Reva, Ian Civil, Felipe Vega, Massimo Chiarugi, Alain Chichom-Mefire, Boris Sakakushev, Andrew Peitzman, Osvaldo Chiara, Fikri Abu-Zidan, Marc Maegele, Mario Miccoli, Mircea Chirica, Vladimir Khokha, Michael Sugrue, Gustavo P. Fraga, Yasuhiro Otomo, Gian Luca Baiocchi, Fausto Catena, the WSES Trauma Quality Indicators Expert Panel

**Affiliations:** 1 General Emergency and Trauma Surgery Department, Pisa University Hospital, Via Paradisa, 2, 56124 Pisa, Italy; 2 Division of General Surgery, Rambam Health Care Campus, Haifa, Israel; 3 Ernest E Moore Shock Trauma Center, Denver Health, Denver, CO USA; 4 Department of Surgery, Harborview Medical Center, University of Washington, Seattle, WA USA; 5 Riverside University Health System, Riverside, CA USA; 6 Division of Trauma and Acute Care Surgery, Fundación Valle del Lili, Cali, Colombia; 7 VCU Medical Center, Richmond, VA USA; 8 General, Acute Care, Abdominal Wall Reconstruction, and Trauma Surgery, Foothills Medical Centre, Calgary, Canada; 9 Department of Trauma and Acute Care Surgery, Scripps Memorial Hospital La Jolla, La Jolla, San Diego, CA USA; 10 General and Emergency Surgery, Macerata Hospital, Macerata, Italy; 11 Department of General and Thoracic Surgery, University Hospital of Giessen, Giessen, Germany; 12 General, Emergency and Trauma Surgery Department, Bufalini Hospital, Cesena, Italy; 13 Abdominal Center, Helsinki University Hospital, Helsinki, Finland; 14 Department of War Surgery, Kirov Military Medical Academy, Saint-Petersburg, Russia; 15 General and Emergency Surgery Dept., Auckland City Hospital, Auckland, New Zealand; 16 Department of Surgery, Hospital Angeles Lomas, Mexico City, Mexico; 17 Faculty of Health Sciences, University of Buea, Buea, Cameroon; 18 Douala Gynaeco-Obstetric and Pediatric Hospital, Douala, Cameroon; 19 General Surgery Department, University Hospital St George, Plovdiv, Bulgaria; 20 Department of Surgery, University of Pittsburgh School of Medicine, Pittsburgh, USA; 21 Trauma Team and General Surgery, ASST Niguarda, Milan, Italy; 22 Department of Surgery, College of Medicine and Health Sciences, UAE University, Al-Ain, United Arab Emirates; 23 Department of Trauma and Orthopedic Surgery, Cologne-Merheim Medical Center (CMMC), University Witten/Herdecke (UW/H), Cologne, Germany; 24 Statistic Dept., Pisa University, Pisa, Italy; 25 Centre Hospitalier Universitaire Grenoble Alpes, Grenoble, France; 26 Department of Emergency Surgery, City Hospital, Mozyr, Belarus; 27 General Surgery Dept., Letterkenny Hospital, Letterkenny, Ireland; 28 Division of Trauma Surgery, School of Medical Sciences, University of Campinas, Campinas, Brazil; 29 Trauma and Acute Critical Care Center, Tokyo Medical and Dental University Hospital, Tokyo, Japan; 30 General Surgery, Brescia University Hospital, Brescia, Italy; 31 Emergency and Trauma Surgery, Parma University Hospital, Parma, Italy

**Correction: World Journal of Emergency Surgery (2021) 16:6** 10.1186/s13017-021-00350-7

Following publication of the original article [1], one of the collaborator names was incorrectly written as “Hossein Samadi Kaf” instead of “Hossein Samadi Kafil” in The WSES Trauma Quality Indicators Expert Panel. The incorrect and correct names are listed in this correction article.

Incorrect author name: Hossein Samadi Kaf

Correct author name: Hossein Samadi Kafil

The original article has been updated.

## References

[1] Coccolini F, Kluger Y, Moore EE, et al. Trauma quality indicators: internationally approved core factors for trauma management quality evaluation. World J Emerg Surg. 2021;16:6. 10.1186/s13017-021-00350-7. 33622373 10.1186/s13017-021-00350-7 PMC7901006

